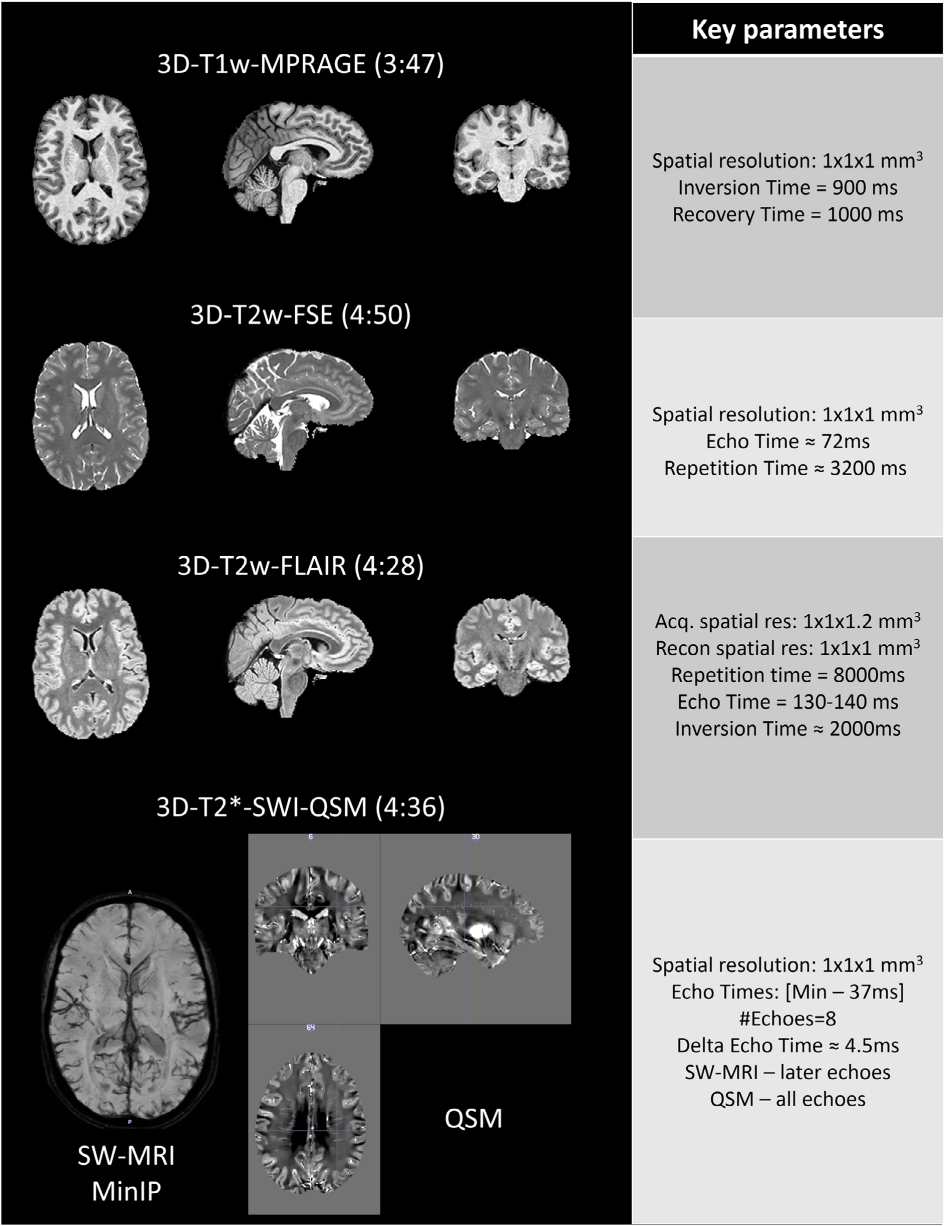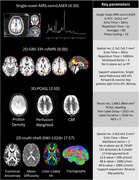# Rational and design of PREDICTOM MRI protocol

**DOI:** 10.1002/alz70856_102571

**Published:** 2025-12-25

**Authors:** Ana Bea Solana Sanchez, Brice Fernandez, David J Lythgoe, Flavio Dell'Acqua, Fernando O Zelaya, Steven CR Williams, Ashwin V Venkataraman, Maarten Naeyaert, Peter Van Schuerbeek, Hubert Raeymaekers, Ralph Noeske, Julie Poujol, Brian Burns, David Shin, Rachid Mahdjoub, Suchandrima Banerjee, PREDICTOM Consortium

**Affiliations:** ^1^ GE Healthcare, Munich, Bayern, Germany; ^2^ Institute of Psychiatry, Psychology and Neuroscience, King's College London, London, United Kingdom; ^3^ GE HealthCare, Buc, Ile‐de‐France, France; ^4^ Institute of Psychiatry, Psychology and Neuroscience,, King's College London, London, United Kingdom; ^5^ Universitair Ziekenhuis Brussel (UZ Brussel), Brussels, Belgium; ^6^ GE HealthCare, Berlin, Germany; ^7^ GE HealthCare, Buc, France; ^8^ GE HealthCare, Seattle, WA, USA; ^9^ GE HealthCare, Menlo Park, CA, USA

## Abstract

**Background:**

The PREDICTOM (Prediction of Neurodegenerative Disease using an AI driven Screening Platform – www.predictom.eu) project is an EU funded project aimed at developing new biomarkers to identify people at high risk of developing Alzheimer Disease (AD) (1).

Here, we aim to describe the rationale behind the MR protocol that will be used at the seven different clinical centers across Europe in the study.

**Method:**

The PREDICTOM study will collect MR data from 615 subjects (target ratio: 80/20% at higher/lower risk). The targeted population will be healthy and over 50 years old. Seven clinical centers across Europe equipped with 3T MR scanners from two different vendors and six different MR models will acquire the MR data.

Learning from ADNI4 (2), the PREDICTOM MR protocol is designed to target only modern clinical MR scanners. It is intended to be a comprehensive protocol that allows extracting the most promising anatomical and functional features for AD.

As PREDICTOM is a multicenter study, the protocol must be harmonized to reduce the bias across centers/scanners to a reasonable level. The total exam time will not exceed 1 hour including the subject positioning.

**Result:**

PREDICTOM MRI protocol contains eight different datatypes. Figure 1 and Figure 2 contain the main parameters and exemplary images in one healthy volunteer scanned in one of the PREDICTOM clinical centers. The choice of QSM was driven by its performance to better detect microbleeds (3). The MRS acquisition has been included due to consistent evidence of alterations in N‐acetylaspartate and myo‐Inositol in early stages of AD (4). For scanner with lower gradient performance, the rsfMRI protocol might use slightly partial Fourier and the multi‐shell diffusion will be reduced to 2‐shell protocol, excluding b=2000.

**Conclusion:**

The protocol has been tested on 4 scanners (2 vendors, 3 models) and the harmonisation is still in progress. The PREDICTOM MR protocol was designed to allow the exploration of existing and novel MRI biomarkers for early identification of people at high risk of developing AD.

**References**

1. Brem AK, AAIC 2024.

2. Arani A, Alzheiemer´s & dementia 2024.

3. Lee K, Neuroimaging 2023.

4. Maul S, Frontiers in Psychiatry 2020.